# Development of a highly sensitive TaqMan method based on multi-probe strategy: its application in ASFV detection

**DOI:** 10.1093/biomethods/bpae011

**Published:** 2024-02-19

**Authors:** Shuxiang Ding, Tianren Shen, Zixuan Feng, Sujing Diao, Yan Yan, Zhenkun Du, Yulan Jin, Jinyan Gu, Jiyong Zhou, Min Liao, Weiren Dong

**Affiliations:** MOA Key Laboratory of Animal Virology, Zhejiang University Center for Veterinary Sciences, Hangzhou 310058, China; College of Animal Sciences, Zhejiang University, Hangzhou, 310058, China; College of Animal Sciences, Zhejiang University, Hangzhou, 310058, China; College of Animal Sciences, Zhejiang University, Hangzhou, 310058, China; MOA Key Laboratory of Animal Virology, Zhejiang University Center for Veterinary Sciences, Hangzhou 310058, China; College of Animal Sciences, Zhejiang University, Hangzhou, 310058, China; MOA Key Laboratory of Animal Virology, Zhejiang University Center for Veterinary Sciences, Hangzhou 310058, China; College of Animal Sciences, Zhejiang University, Hangzhou, 310058, China; Zhoushan City Bureau of Agriculture and Rural Development, Zhejiang 316000, China; MOA Key Laboratory of Animal Virology, Zhejiang University Center for Veterinary Sciences, Hangzhou 310058, China; College of Animal Sciences, Zhejiang University, Hangzhou, 310058, China; MOA Key Laboratory of Animal Virology, Zhejiang University Center for Veterinary Sciences, Hangzhou 310058, China; College of Animal Sciences, Zhejiang University, Hangzhou, 310058, China; MOA Key Laboratory of Animal Virology, Zhejiang University Center for Veterinary Sciences, Hangzhou 310058, China; College of Animal Sciences, Zhejiang University, Hangzhou, 310058, China; MOA Key Laboratory of Animal Virology, Zhejiang University Center for Veterinary Sciences, Hangzhou 310058, China; College of Animal Sciences, Zhejiang University, Hangzhou, 310058, China; MOA Key Laboratory of Animal Virology, Zhejiang University Center for Veterinary Sciences, Hangzhou 310058, China; College of Animal Sciences, Zhejiang University, Hangzhou, 310058, China

**Keywords:** qPCR, multi-probe, TaqMan, water sample, ultrafiltration, African swine fever virus

## Abstract

The establishment of high sensitive detection method for various pathogenic microorganisms remains constantly concerned. In the present study, multi-probe strategy was first systematically investigated followed by establishing a highly sensitive TaqMan real-time fluorescent quantitative PCR (qPCR) method for detecting African swine fever virus (ASFV). Briefly, four probes based on the *B646L* gene of ASFV were designed and the effects of different combinations of the probes in a single TaqMan qPCR assay on the detection sensitivity were investigated. As less as 0.5-5 copies/μl of the ASFV gene was detected by the established TaqMan qPCR assay. Furthermore, plasmid harboring the *B646L* in water samples could be concentrated 1000 times by ultrafiltration to enable a highly sensitive detection of trace viral nucleic acids. Moreover, no cross-reactivity was observed with other common clinical swine viruses such as PCV2, PCV3, PCV4, PEDV, PDCoV, CSFV, PRRSV, and PRV. When detecting 173 clinical porcine serum samples, the coincidence rate between the developed method and WOAH (World Organization of Animal Health) recommended method was 100%. This study might provide an integrated strategy to achieve higher detection sensitivity of trace pathogenic microorganisms and applicably sensitive TaqMan-based qPCR assays.

## Graphical Abstract

**Graphical Abstract:**
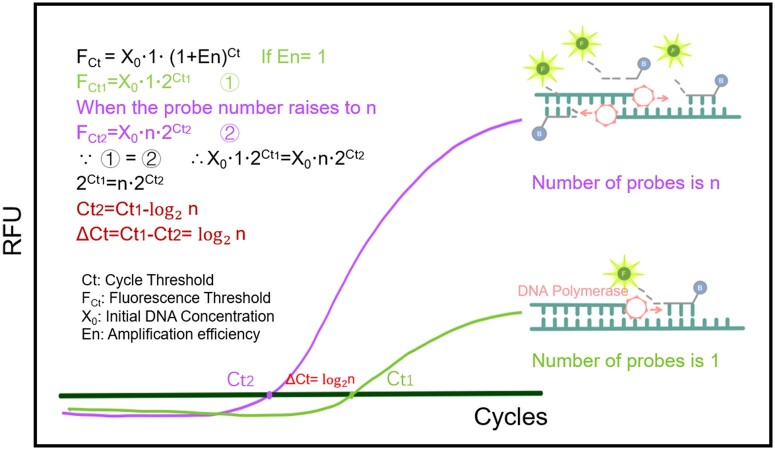

## Introduction

The real-time fluorescent quantitative PCR (qPCR) works by adding nucleic acid dye (SYBR Green) that can bind to the double-stranded DNA or free fluorescent moiety produced by 5′–3′ exonuclease activity of Taq enzyme (TaqMan) to the PCR reaction, followed by detecting real-time fluorescent signal accumulation and conducted absolute or relative analysis of the initial copy quantity of the target gene [1]. The qPCR is ubiquitously utilized for quantitative or qualitative detection of specific gene abundance because of its specificity and simplicity. However, the SYBR Green-based qPCR cannot completely exclude the non-specific PCR amplification of double strand DNA which could bind to the dye, followed by causing decreased Ct value or even false positive. Meanwhile, TaqMan qPCR with additional sequence-specific probe has the advantage of accuracy as well as multiplex detection. Accordingly, TaqMan qPCR is universally involved in the frontline detection and has a variety of applications in early disease diagnosis, food pathogenic microbial detection, drug research, animal disease monitoring [2–6].

Multiple factors affect the sensitivity of TaqMan qPCR assay, including the enzyme type, primer sequence, probe sequence, component ratio, PCR amplification parameter, etc. [7]. For example, Arif *et al*. modified the primers by adding a customized 22-nucleotide long tail at the 5′ terminus and effectively improved the detection sensitivity [8]. Terry *et al*. used two detection systems in combination with three different detection chemistries and finally determined the optimal combination quantitative detection of genetically modified organisms [9]. Meanwhile, nucleic acids enrichment strategy is usually required for determinating trace amount of pathogen with concentration lower than the detection limit of the PCR. The ethanol precipitation is one of the most well-known methods for chemical enrichment [10] which may lead to loss of nucleic acids. Moreover, ultrafiltration is a membrane-based separation technique that capitalizes the microporous structure of a semi-permeable membrane to achieve selective separation and recovery of substances [11], implying the involvement of ultrafiltration in nucleic acid enrichment might be beneficial for the improvement of the detection sensitivity of qPCR.

Notably, in terms of the principle of TaqMan PCR, when the number of fluorescent moiety released from the probe increased in each PCR cycle, the generated and accumulated fluorescence signal should be doubled or even multiplied, theoretically reducing the Ct value and improving the detection sensitivity. However, few studies had been conducted to reveal this speculation. To our knowledge, only one published paper investigated the effect of probe numbers (up to three probes) on the detection sensitivity and finally found that the dual-probe increased the detection sensitivity, however, triple-probe did not further enhance the sensitivity but increased the variability between duplicate readings [12]. Meanwhile, Nagy *et al*. used two identically labeled hydrolysis probes in single assay to prevent false negativity due to probe binding failure [13]. Accordingly, it seems that the maximum number of probes can only be two to improve the detection sensitivity. As mentioned above, the fluorescence signal produced by a target gene can theoretically be doubled or even multiplied if the number of probes increased, leading to the decrease of the Ct value and increase of the detection sensitivity. To reveal the relationship between theoretical and practical results, it is worthy to systematically compare the effects of single-probe, double-probe, triple-probe, and even quadruple-probe on the sensitivity of TaqMan qPCR taking the detection of the African swine fever virus (ASFV) as an example.

African swine fever (ASF), caused by ASFV, is a highly contagious disease with a fatality rate of up to 100% in domestic pigs and wild boars [14]. ASFV has evolved the ability to manipulate host immune responses by encoding many immune escape genes. Therefore, despite extensive study on various vaccine approaches, there are still no potential vaccine candidates [15, 16]. Therefore, effective detection of ASFV is critical for disease control and surveillance. The TaqMan qPCR, recommended by the World Organization for Animal Health (WOAH) [17], had a limit of detection (LOD) of 10–100 copies/μl which was approximately six times of that of conventional PCR [18]. This study focused on the common question of whether the sensitivity of TaqMan qPCR can be further improved and whether multiple probes (≥3) had higher sensitivity than dual-probe. Briefly, we designed multiple probes according to the ASFV *B646L* gene to investigate whether multiple probes are beneficial to enhance the sensitivity of TaqMan qPCR, with the aim of improving the sensitivity of single-target gene detection. As pigs could be also infected by low concentration ASFV from the virus-contaminated water [19], detection of the virus in water sample where the virus abundance is lower than LOD is still challengeable. This experiment aimed to establish a TaqMan qPCR method for highly sensitive detection of ASFV in environmental and clinical samples with nucleic acid ultrafiltration enrichment as well as multi-probe strategy. It may provide a new idea for establishing a highly sensitive detection method of ASFV and even other pathogens.

## Materials and methods

### Viral genome and plasmid

The *B646L* gene of ASFV genotype II strain was synthesized by Zhejiang Sunya Biotechnology Co., Ltd China according to a published sequence (GenBank: MK333180.1), and cloned into the pUC57 to obtain the plasmid pUC57-B646L. The *E.coli* JM109 containing the recombinant plasmid was cultured and the plasmid DNA was extracted (Tiangen, Plasmid Small Extraction Kit, DP103-02) for further study. The viral nucleic acid of porcine circovirus type 2 (PCV2), porcine circovirus type 3 (PCV3), porcine circovirus type 4 (PCV4), porcine epidemic diarrhea virus (PEDV), porcine delta coronavirus (PDCoV), classical swine fever virus (CSFV), porcine reproductive and respiratory syndrome virus (PRRSV), porcine pseudorabies virus (PRV) were purified and stored at the Key Laboratory of Animal Virology, Ministry of Agriculture, Zhejiang University.

### Primers and probes design

**Figure 1.**
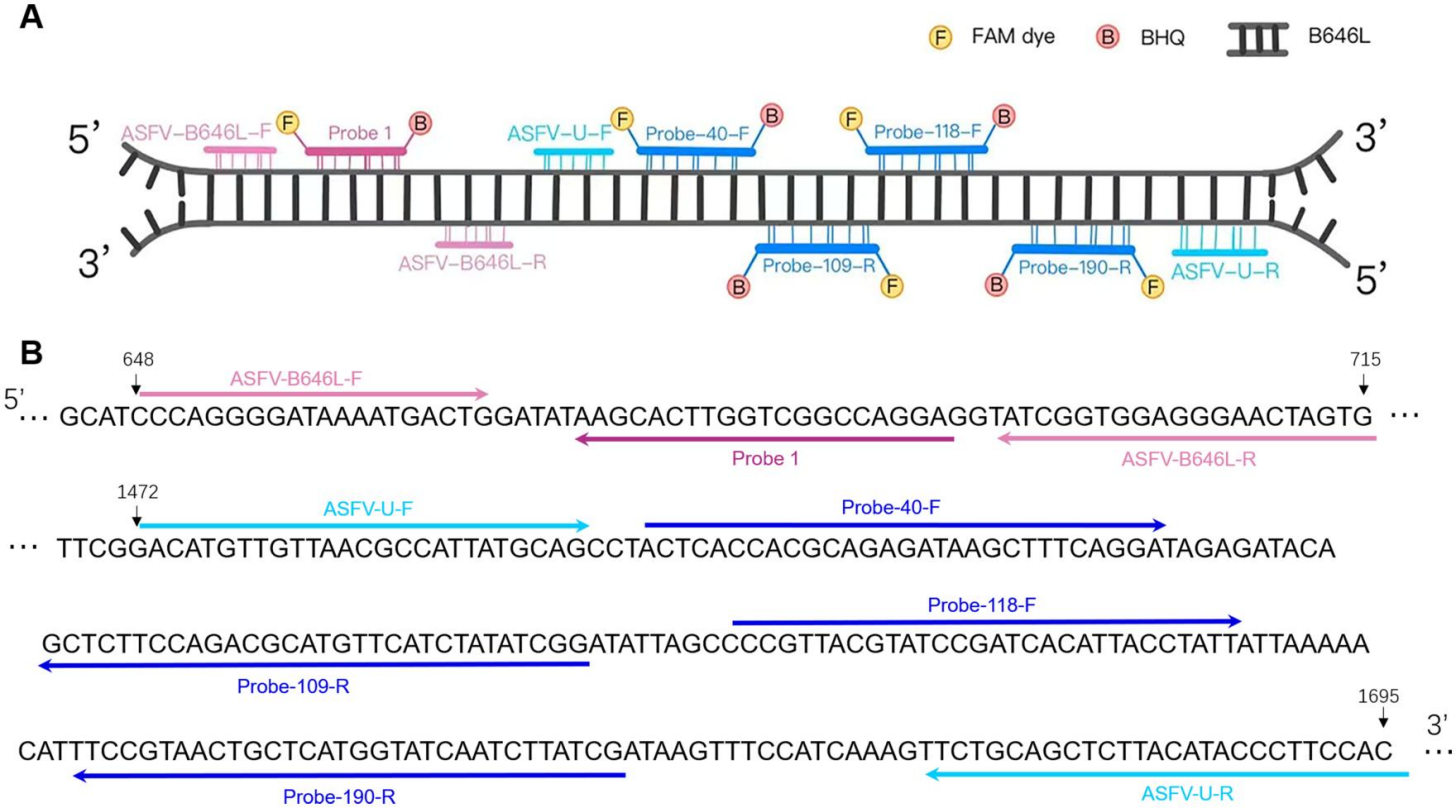
The diagram of primers and probes for detecting *B646L* of ASFV. (**A**) Relative positions of different primers and probes. (**B**) The sequences of primers and probes.

The primers and probes of TaqMan qPCR were designed according to the conserved ASFV genotype II sequence. Four probes targeting *B646L* gene were labeled with reporter dye 6-carboxyfluorescein (FAM) and the 3′-quencher BHQ1. The positions of the specific primers and probes targeting to the *B646L* gene were shown in Fig. 1. The information of primers and probes were listed in Table 1. The TaqMan qPCR reaction with universal primers ASFV-B646L-F, ASFV-B646L-R and probe ASFV-B646L-probe 1 (Table 1) for the detection of ASFV recommended by WOAH were carried out as control.

**Table 1.** Probes and primer sequences used in the TaqMan qPCR assay.

Name	Sequence (5′–3′)	Resource
ASFV-B646L-F	CCCAGGRGATAAAATGACTG	WOAH
ASFV-B646L-R	CACTRGTTCCCTCCACCGATA	WOAH
ASFV-B646L-probe 1	FAM-TCCTGGCCRACCAAGTGCTT-BHQ1	WOAH
ASFV-U-F	GACATGTTGTTAACGCCATTATGCAG	This study
ASFV-U-R	GTGGAAGGGTATGTAAGAGCTGCAGA	This study
ASFV-probe-40-F	FAM-ACTCACCACGCAGAGATAAGCTTTCAGGA-BHQ1	This study
ASFV-probe-109-R	FAM-CCGATATAGATGAACATGCGTCTGGAAGAGC-BHQ1	This study
ASFV-probe-118-F	FAM-CCCGTTACGTATCCGATCACATTACCTATT-BHQ1	This study
ASFV-probe-190-R	FAM-CGATAAGATTGATACCATGAGCAGTTACGGAA-BHQ1	This study

### Establishment and optimization of TaqMan qPCR assay

The experiments were divided into four groups based on the numbers of the probes as listed in the Table 2. TaqMan qPCR reaction of quadruple-probe group contained 10.0 μl of qPCR Probe Master Mix (Vazyme AceQ^®^, Q122-02), 10.0 μM of the primers (ASFV-U-F and ASFV-U-R), 10.0 μM of the probes (ASFV-probe-40-F, ASFV-probe-109-R, ASFV-probe-118-F, ASFV-probe-190-R), 2.0 μl of template and ddH_2_O to a final volume of 20.0 μl. Triple-probe, double-probe and single-probe PCR reactions altered the number of probes on the basis of quadruple-probe, and detailed probe combinations were shown in Table 2. The TaqMan-based PCR program was as follows: pre-denaturation at 95°C for 5 min, and 40 amplification cycles of 95°C for 10 s and 60°C for 30 s (Bio-Rad, CFX 96 touch). Furthermore, the TaqMan qPCR reaction was optimized as follows: first, the concentrations of the primers and probes (1.0, 5.0, 10.0, and 15.0 μM) were screened by the matrix method. Second, different annealing temperatures, including 55.0, 55.6, 56.8, 60.7, 62.5, 63.5, and 64.0°C, respectively, were screened to confirm the optimum amplification conditions. The optimized PCR reaction condition was selected for the subsequent experiments.

**Table 2.** Effect of different probe numbers on the Ct value of TaqMan qPCR assay.

Number of Probe	Name of Probe	Ct value	Average Ct
Single-probe	probe 40	25.39	25.26 ± 0.46
	probe 109	24.80	
	probe 118	25.09	
	probe 190	25.77	
Double-probe	probe 40/109	24.07	23.97 ± 0.65
	probe 40/118	23.40	
	probe 40/190	24.00	
	probe 109/118	23.15	
	probe 109/190	24.62	
	probe 118/190	24.60	
Triple-probe	probe 40/109/118	23.17	23.37 ± 0.37
	probe 40/118/190	23.42	
	probe 40/109/190	23.74	
	probe 109/118/190	23.14	
Quadruple-probe	probe 40/109/118/190	23.31	23.31

### Standard curve and enrichment test

The copy number of plasmid pUC57-B646L was determined by digital PCR (Bio-Rad, Droplet Digital PCR, QX200), and then the plasmid was diluted to obtain a series of concentration gradient from 5 × 10^6^ to 5 × 10° copies/μl. Two microliter of each dilution was taken as template and the gene was amplified according to the optimal condition of TaqMan triple-probe PCR assay as mentioned above. The copy number of each reaction was proportional to the threshold cycle (Ct) of dilution, and the standard curve of ASFV-B646L was established based on this relationship.

To evaluate the enrichment of virus, the positive template was diluted with ddH_2_O to 300 ml with 0.001 copies/μl, and 900 μl of the dilution was used as non-concentrated (NC) sample. The diluted samples were divided into three groups, which were concentrated by 100, 500, and 1000 times, respectively. In detail, 15 ml sample solution was added to centrifugal filter (Merck Millipore, UFC901096) and centrifuged at 4000×*g* for about 40 min until the sample concentrated to finally 150 μl (100 times concentrated) and marked as C-100. Similarly, 150 μl of 500 times and 1000 times concentrated samples were prepared, and marked as C-500 and C-1000, respectively.

### Sensitivity test

The standard plasmid pUC57-B646L with an initial concentration of 5 × 10^6^ copies/μl was diluted 10 times to 5 × 10^−2^ copies/μl by ddH_2_O. Amplification was performed according to the optimal reaction condition as mentioned previously, and 2.0 μl of each dilution sample was used as the PCR template to calculate the LOD of the triple-probe 40/109/118 TaqMan qPCR assay.

Furthermore, the enriched samples were also tested for sensitivity. The experiments were divided into the control group without templates and the experimental groups with templates of different concentrations. The PCR template of the experimental groups was NC, C-100, C-500, C-1000, and positive samples as positive controls. For each sample, the qPCR were carried out for three replicates per group. According to the results, the detection limit and sensitivity of triple-probe 40/109/118 TaqMan qPCR assay for the detection of ASFV in water was analyzed.

### Specificity test

The purified nucleic acids of the PCV2, PCV3, PCV4, PEDV, PDCoV, CSFV, PRRSV, and PRV were used to evaluate the specificity of the triple-probe 40/109/118 TaqMan PCR detection method, pUC57-B646L was used as the positive control, and ddH_2_O was used as the negative control. Nucleic acids concentrations were determined by ultra-micro spectrophotometer (Thermo Fisher Scientific, NanoDrop One) and the copy numbers of the nucleic acids were calculated based on the molecular weight and Avogadro's number.

### Reproducibility test

Plasmids with four different dilution concentrations were used as PCR templates, and each dilution was repeated for three times under the same conditions to conduct the intra-group repeatability. Each dilution was repeated for three times at different time points under the same condition to conduct the inter-group repeatability. The standard deviation and coefficient of variation (CV) that indicates the size of a standard deviation in relation to its mean were calculated by repeated detection of each concentration for three times and three rounds.

### Clinical sample test

One hundred and seventy three blood serum samples from pigs were collected from 2 farms in Zhejiang Province. After extracting nucleic acids (Takara, Virus Nucleic Acid Extraction Kit, TKR-9766) from these clinical samples suspected of ASFV infection, the triple-probe 40/109/118 TaqMan PCR detection system and the single-probe 109 detection system were used for clinical detection. Meanwhile, the coincidence rate of positive samples was verified with WOAH recommended detection system to evaluate the feasibility of this method.

## Results

### Effect of different number of probes on Ct value of TaqMan qPCR

**Figure 2.**
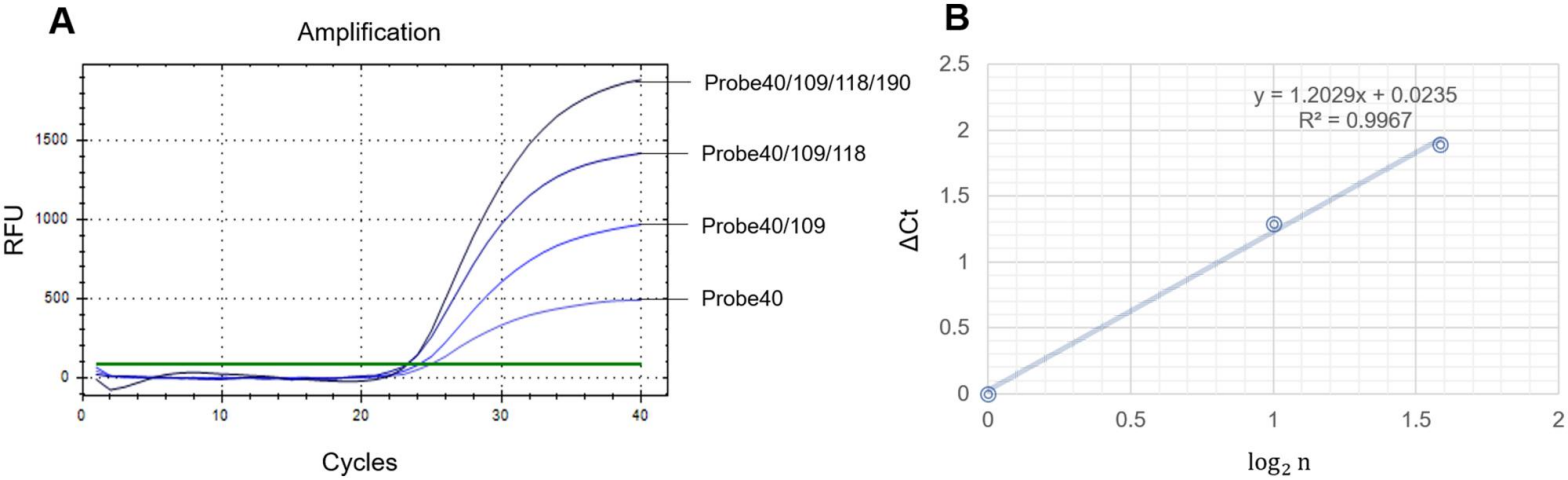
Fluorescence quantitative PCR amplification curves of different number of probes and the linear relationship. (**A**) Selecting representative curves of four TaqMan qPCR reactions which were based on the optimal reaction system and amplification reaction conditions. (**B**) The photography depicts linear relationship between average ΔCt and $\log_{2}n$ value. The coefficient of determination (*R*^2^) and equation of the regression curve (*y*) were calculated.

As shown in Table 2 and Fig. 2, the Ct values of the TaqMan qPCR were corresponding to different probe combinations. The average Ct values of the single-probe, double-probe, triple-probe and quadruple-probe were 25.26, 23.97, 23.37, and 23.31, respectively. Although the Ct values of the double-probe varied according to different combinations, the average Ct values of double-probe (23.97) was 1.29 less than that of the single-probe (25.26). The average Ct value of the triple-probe (23.37) was 1.89 and 0.60 smaller than that of the single-probe (25.26) and double-probe (23.97), respectively, which was basically consistent with the theoretical value. However, when the numbers of probes were increased to four, the Ct value of quadruple-probe decrease to 23.31 which was 1.95, 0.66 and 0.06 less than the average Ct value of single-probe, double-probe and triple-probe, respectively. Collectively, the Ct value decreased as expected while the number of probe raised as shown in the Fig. 2A. Notably, the values of $\log_{2}n$ (*n* < 4) and the corresponding ΔCt presented good linear relationship (*R*^2^ = 0.9967, Fig. 2B).

### Optimization of TaqMan qPCR reaction assay

According to the results of primer concentration matrix method and temperature gradient assays, the amplification efficiency was the highest when the primer concentration was 10.0 μM, the probe concentration was 15.0 μM (Supplementary Table S1) and the annealing temperature was 62.5°C (Supplementary Table S2). The optimal reaction condition for triple-probe TaqMan qPCR contained 10.0 μl of qPCR Probe Master Mix, 10.0 μM of the primers (ASFV-U-F and ASFV-U-R), 15.0 μM of the probes (ASFV-probe-40-F, ASFV-probe-109-R, ASFV-probe-118-F), 2.0 μl of template and ddH_2_O to a final volume of 20.0 μl. The cycling conditions were 95°C for 5 min, 40 cycles of 95°C for 10 s and 62.5°C for 30 s.

### Standard curve

**Figure 3.**
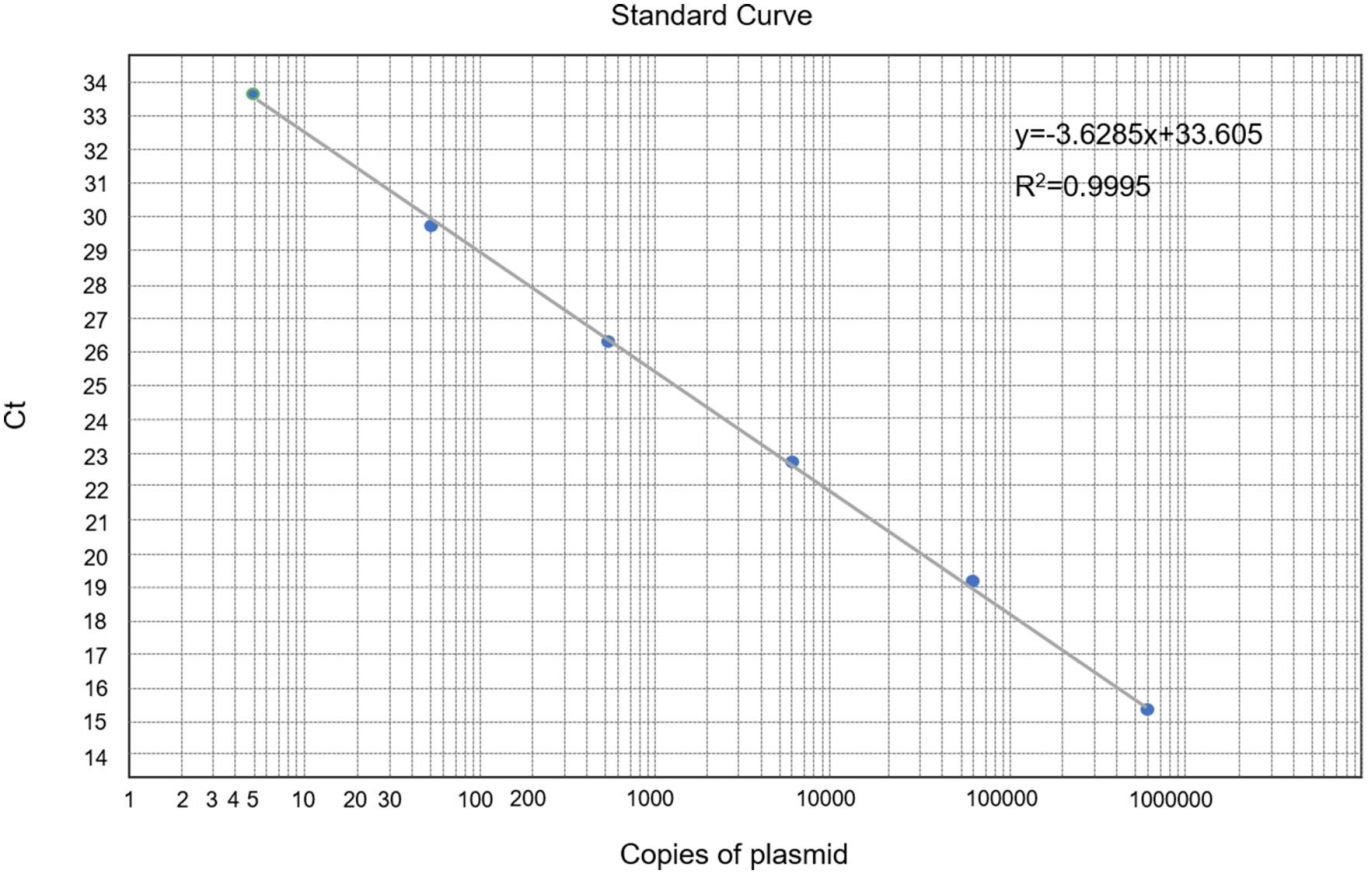
Standard curves of the triple-probe 40/109/118 TaqMan qPCR assay. The dynamic range and detection limit were evaluated by testing 10-fold serial diluted of plasmid (from 5.0 × 10^6^ to 5.0 × 10° copies/μl) against the Ct value. The coefficient of determination (*R*^2^) and equation of the regression curve (*y*) were calculated.

The standard curve was drawn according to the Ct value and the corresponding copy number (Fig. 3). The standard curve was Y = −3.6285X + 33.605 (Y: Ct value, X: copies of plasmid), and the correlation coefficient (*R*^2^) was 0.9995. According to the standard curve, the linear relationship of each reaction was good in the range of 5 × 10^6^ – 5 × 10° copies/μl.

### Sensitivity

**Figure 4.**
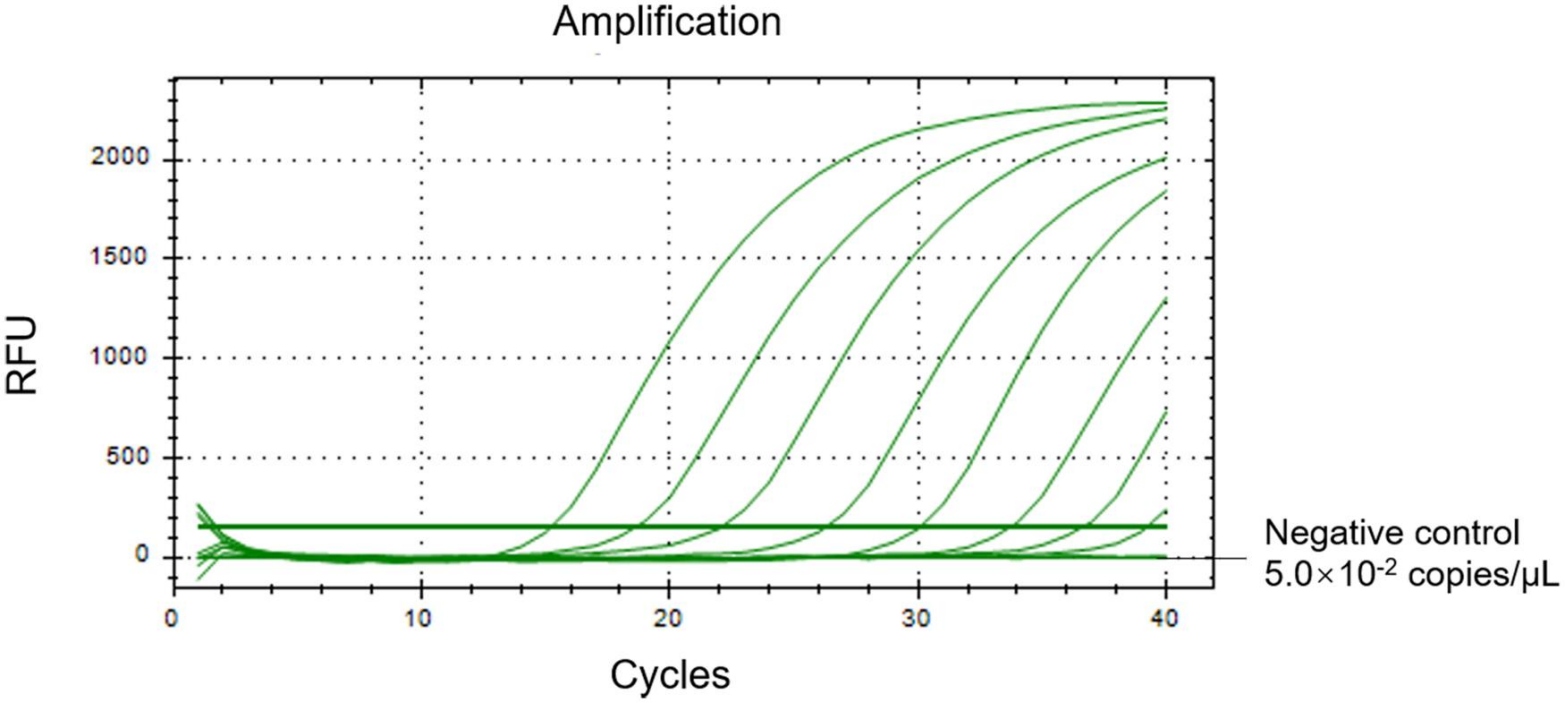
Sensitivity test curves of triple-probe 40/109/118 TaqMan qPCR assay. The dynamic curves were generated by using the plasmid final concentration ranged from 5.0 × 10^6^ to 5.0 × 10^−2^ copies/μl.

Serial dilutions of the standard plasmid (from 5.0 × 10^6^ to 5.0 × 10^−2^ copies) were used as templates to detect LOD of the triple-probe 40/109/118 TaqMan qPCR method. The results showed that LOD of this method was 5.0 × 10^−1^ copies/μl (Fig. 4). Moreover, the Ct values of qPCR of triple-probe 40/109/118 were smaller than that of the single-probe 109 (Supplementary Fig. S1). When conducting the enrichment assay of viral nucleic acid in water sample with ultrafiltration, the reality was slightly different from the expectation. In detail, it was expected that the 15 ml solution could be centrifuged once to enrich into 150 μl solution. In the actual process, the volume of concentrated samples less than 150 μl after concentration were supplemented to 150 μl by adding ddH_2_O. As shown in Table 3, the initial sample NC and C-100 could not be detected, while C-500 and C-1000 could be detected positively after 500 and 1000 times enrichment.

**Table 3.** Triple-probe 40/109/118 TaqMan qPCR results of ASFV enrichment experiment in water.

Sample name	Enrichment ratio	Theoretical concentration (copies/μl)	Ct value
NC	0	0.001	–
C-100	100	0.1	–
C-500	500	0.5	38.74 ± 0.33
C-1000	1000	1	37.08 ± 0.36

### Specificity

**Figure 5.**
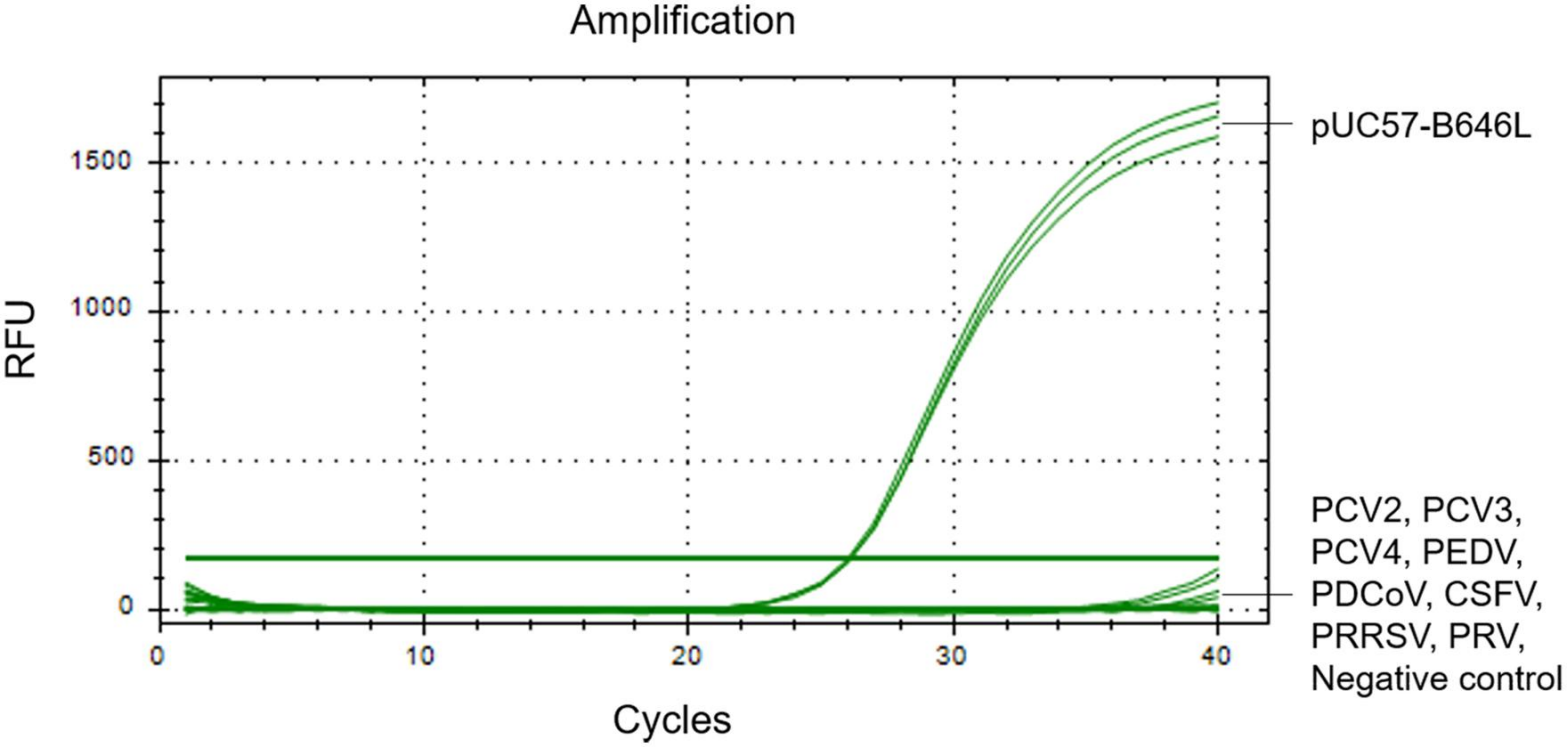
Specificity of the triple-probe 40/109/118 TaqMan qPCR assay. The assay was used to amplify pUC57-B646L, PCV2, PCV3, PCV4, PEDV, PDCoV, CSFV, PRRSV, PRV. The X-axis represents the cycles, and the Y-axis represents the fluorescence data.

Nucleic acids of PCV2 (2.09 × 10^7^ copies/μl), PCV3 (2.21 × 10^7^ copies/μl), PCV4 (9.94 × 10^7^ copies/μl), PEDV (4.17 × 10^7^ copies/μl), PDCoV (1.08 × 10^7^ copies/μl), CSFV (1.48 × 10^7^ copies/μl), PRRSV (1.67 × 10^7^ copies/μl), and PRV (7.18 × 10^7^ copies/μl) were used as qPCR templates, pUC57-B646L standard plasmid (5 × 10^3^ copies/μl) as a positive control, and ddH_2_O as a negative control. The results showed that fluorescence signal could be observed only with pUC57-B646L but no with nucleic acids of PCV2, PCV3, PCV4, PEDV, PDCoV, CSFV, PRRSV, and PRV (Fig. 5), indicating that the established triple-probe 40/109/118 TaqMan qPCR method was specific for ASFV without any cross-reactivity to detected swine viruses.

### Reproductivity

In order to evaluate the repeatability of qPCR, standard plasmids in four different concentrations (Table 4) were used as templates for intra- and inter-assay comparisons. The results showed that the coefficient of variation of intra-assay were 0.57%, 0.38%, 0.53%, and 1.00%, and the results showed that the coefficient of variation of inter-assay were 0.53%, 0.37%, 0.10%, and 0.83%.

**Table 4.** Intra- and Inter-assay reproducibility of the triple-probe 40/109/118 TaqMan qPCR assay.

Different dilution (copies/μl)	Intragroup repeatability			Intergroup repeatability		
	Meant Ct	SD	CV (%)	Meant Ct	SD	CV (%)
5 × 10^6^	15.21	0.09	0.57	15.14	0.08	0.53
5 × 10^4^	22.26	0.08	0.38	22.33	0.08	0.37
5 × 10^2^	29.68	0.16	0.53	29.53	0.30	0.10
5 × 10^0^	36.38	0.36	1.00	36.48	0.30	0.83

### Clinical sample detection

The clinically collected serum samples were detected using the established triple-probe 40/109/118 and single-probe 109 TaqMan qPCR, with pUC57-B646L standard plasmid as a positive control and ddH_2_O as a negative control. The results were shown in Table 5. Among 173 clinical samples, 137 negative samples and 36 positive samples were detected by the triple-probe 40/109/118 TaqMan qPCR method, with a positive rate of 20.81%; while 143 negative samples and 30 positive samples were detected by the single-probe TaqMan qPCR method, with a positive rate of 17.34%. Meanwhile, 137 negative samples and 36 positive samples were also detected according to the WOAH recommended method, which was in 100% correspondence with the triple-probe 40/109/118 TaqMan qPCR (Supplementary Table S3).

**Table 5.** Ct value of single-probe 109 and triple-probe 40/109/118 TaqMan qPCR of positive clinical samples.

Sample number	Ct of single-probe PCR	Ct of triple-probe PCR	ΔCt	Sample number	Ct of single-probe PCR	Ct of triple-probe PCR	ΔCt
1	–	37.52	>2.48	19	34.05	32.62	1.43
2	37.48	35.21	2.28	20	33.69	31.72	1.97
3	37.09	34.56	2.53	21	34.31	32.50	1.81
4	34.94	33.27	1.68	22	29.89	28.14	1.75
5	38.75	36.16	2.59	23	38.28	38.20	0.08
6	36.43	34.13	2.31	24	36.43	34.55	1.88
7	34.51	32.74	1.77	25	34.44	32.57	1.87
8	36.74	35.20	1.54	26	39.15	38.84	0.31
9	33.34	32.21	1.13	27	36.51	34.23	2.28
10	34.98	33.16	1.82	28	38.84	35.30	3.54
11	–	38.38	>1.62	29	–	38.70	>1.30
12	38.60	32.50	6.10	30	–	39.11	>0.89
13	–	39.24	>0.76	31	38.53	35.77	2.76
14	38.58	36.06	2.53	32	37.21	34.55	2.66
15	30.05	27.74	2.31	33	34.04	31.98	2.06
16	38.83	37.88	0.95	34	–	39.47	>0.53
17	38.43	37.75	0.68	35	39.63	34.56	5.07
18	38.79	37.03	1.76	36	28.25	26.36	1.89

Ct ≥ 40 was considered negative (-).

## Discussion

For many pathogenic diseases that have not yet effective vaccines and therapeutic drugs, diagnosis of pathogens is the first and critical element for adopting effective disease control strategies. TaqMan qPCR has emerged as a preferred frontline clinical method for detecting viral or bacterial pathogens in human, animal, and plant specimens due to its exceptional sensitivity and specificity within the realm of diagnostic microbiology [13]. Nonetheless, false-negative diagnosis would occur when the initial copy number of the target gene is extremely low, presenting a potential pathogen-related hazard has gone undetected [20]. Consequently, researchers have diligently sought solutions to improve assay sensitivity and mitigate the occurrence of false negative during testing.

The sensitivity of TaqMan qPCR assay is influenced by various factors, notably, it is theoretically expected to generate a lower Ct value by increasing the number of probes based on the fundamental principles of TaqMan-based PCR. To explore this issue, we systematically compared the effects of different number of probe combinations on detection sensitivity of TaqMan qPCR assay. The results of single-probe study (Table 2) showed that the Ct values varied with different single-probe sequences. Notably, when the probe 190 was utilized in the single-probe qPCR assay (probe 190) or double-probe qPCR assay (probe 40/190, probe 109/190, probe 118/190) or triple-probe qPCR assay (probe 40/118/190, probe 40/109/190), the Ct values exhibited overall higher than those of other corresponding probe combinations. These results suggested that the probe sequence had an influence on the detection sensitivity which was consistent with the findings reported by Nagy *et al*. [13].

Meanwhile, double-probe TaqMan qPCR assays exhibited that different combinations of two probes also had different Ct values. For instance, the Ct values of the probe 109/190 and probe 109/118 were 24.62 and 23.15, respectively, furtherly underscoring the significance of optimization of probe combination. Furthermore, the average Ct value of the six double-probe combinations was approximately 1.30 lower than that of the single-probe assays, indicating that two different probes in a single assay could effectively improve the detection sensitivity. To our knowledge, Yip *et al*. first investigated the impact of different numbers of probes (up to three probes) on the sensitivity of SARS-CoV-2 detection and found that double-probe but not triple-probe could enhance detection sensitivity, implying that one and only one strategy viz. double-probe can improve detection sensitivity by adding the numbers of probes [12]. However, our results revealed that triple probes in a single assay exhibited stronger fluorescence intensity (Fig. 2) and the average Ct value for the triple-probe configuration was 1.72 and 0.60 lower than those of the single-probe and double-probe, suggesting an approximate 3- and 1.5-fold improvement in detection sensitivity as theoretically expected. These results first revealed that the increasing numbers of probes to three could still enhance the fluorescence intensity and reduced the Ct value which was really unexpected when compared with that of the previous report [12]. Notably, the Ct value of quadruple-probe was lowest among the all tested combinations, suggesting the fluorescence intensity could be further accumulated in the quadruple-probe assay. However, an anticipated reduction of Ct value was not observed in the quadruple-probe assay, demonstrating a potential interference within the amplification reaction or probe internal interaction which was worthy to be further investigated or optimized in the near future. Consequently, regarding to the perspective of cost performance, triple-probe seemed to be the most suitable probe numbers in the TaqMan qPCR assay in the present study and could be adopted for subsequent detection of ASFV or even other pathogenic microorganisms.

Accordingly, our experimental results showed that multi-probe strategy could be utilized to establish a highly sensitive TaqMan qPCR method for detecting target genes, and triple-probe or even quadruple-probe which may vary from gene to gene might be beneficial to improve the detection sensitivity. Moreover, if the target gene had mutation(s) in probe sequence, it may lead to decrease probe binding or even completely abolish probe binding [21, 22]. Undoubtedly, it is almost impossible for up to three or even more sequences to mutate simultaneously. Consequently, multi-probe strategy could achieve the utmost minimization of the occurrence of false negative and ensure the stability of the detection assay, and it should have greater application value in the frontline detection especially in RNA virus detection. By mitigating the limitations of single-probe assay, the multi-probe strategy could effectively offer improved stability and sensitivity in disease diagnoses. Actually, we had also successfully applied such multi-probe strategy into the detections of notorious SARS-CoV-2 and *Bursaphelenchus xylophilus* which breeds disease pine wood nematode (PWN) presenting the most devastating disease of pine trees (data not shown). These findings were helpful to promote the understanding of detection methodology and also provided a great significance to disease control strategies.

Ultrafiltration was extensively applied in the separation of macromolecules and colloidal entities, including enzymes, proteins, and viruses [23, 24]. Notably, previous studies had successfully employed ultrafiltration for varying recovery efficiencies of enveloped viruses in water [25], as well as for microbe concentration in drinking water by reducing the total volume 100 L to 3–5 ml [26]. In the present study, we employed the combined methodology of ultrafiltration enrichment and triple-probe approach to achieve a limit of detection as low as 0.001 copies/µl under 1000-fold enrichment conditions. Although the nucleic acid in water medium but not clinical sample was utilized in the ultrafiltration enrichment assay, the integration of ultrafiltration enrichment and triple-probe strategy might offer valuable insights into greatly improving the sensitivity and practicability of detection, finally, promoting the application of the detection methods.

ASF, a dramatic swine disease, has inflicted substantial damage on the global pig industry, particularly impacting China which is the largest pig producer [27, 28]. In light of its importance, the TaqMan qPCR method detecting the most frequently employed *B646L* gene has been endorsed by the WOAH as the preferred and gold standard technique for ASFV detection [29]. Several studies had developed TaqMan qPCR assays targeting ASFV *B646L* gene with varying sensitivity limits of 32.1 copies/μl, 5.8 copies/μl, and 7.9 copies/μl, respectively [30–32]. In this study, a pair of primer and four specific probes were designed and the best probe combination as well as PCR reaction condition were optimized, followed by establishing a reliable triple-probe TaqMan qPCR method. Experimental results revealed that the triple-probe assay designed for the *B646L* gene exhibited approximately three-fold higher detection sensitivity compared to the single-probe assay, and the generated standard curves demonstrated excellent linearity within the dilution range of 5 × 10^6^ to 5 × 10° copies/μl. Moreover, through water ultrafiltration experiments, detection limit as low as 10^−3^ copies/μl or even lower for dispersed low-copy-number viruses in the environment could be achieved. Accordingly, the multi-probe and ultrafiltration enrichment strategy was conductive to serve the implementation of strict and accurate disease prevention and control.

Furthermore, the established method demonstrated high specificity and exhibited no cross-reactivity with other common pathogens of porcine infectious diseases, including PCV2, CSFV, PRV, PRRSV, PEDV, and PDCoV. Meanwhile, the assay exhibited good reproducibility, and the coefficients of variation within and between groups were both less than 1%. Clinical sample analysis further validated the superior sensitivity of the triple-probe assay compared to the single-probe assay, while the positive rate of clinical detection result aligned with that obtained using the WOAH-recommended assay method. Predictably, the multi-probe strategy with or without ultrafiltration enrichment could be ubiquitously utilized to establish a highly sensitive detection systems for other pathogens.

In conclusion, the multiple-probe strategy for TaqMan qPCR was first systematically investigated in this study, and the triple-probe combined with ultrafiltration enrichment method with highly sensitivity was successfully established for detecting ASFV. The combined strategy was expected to be widely held for rapid differential diagnosis and quantitative analysis of pathogenic microorganisms. The results from this study can help improve the understanding of the infection dynamics and prevalence of various pathogenic microorganisms, thereby providing crucial detection tools for epidemiological surveillance efforts. The versatility and potential applicability of this approach made it a valuable asset in combating infectious diseases and safeguarding public health.

## Supplementary Material

bpae011_Supplementary_Data

## Data Availability

The data underlying this article are available in the article and in its online supplementary material.
