# Supplementary material for: Development of a highly sensitive TaqMan method based on multi-probe strategy: its application in ASFV detection
Source: Biol Methods Protoc. 2024 Feb 19;9(1):bpae011. doi: 10.1093/biomethods/bpae011 (PMC10939455; doi:10.1093/biomethods/bpae011)
Supplement: bpae011_Supplementary_Data [file bpae011_supplementary_data.zip › Appendix 1.pdf]

Fig. S1

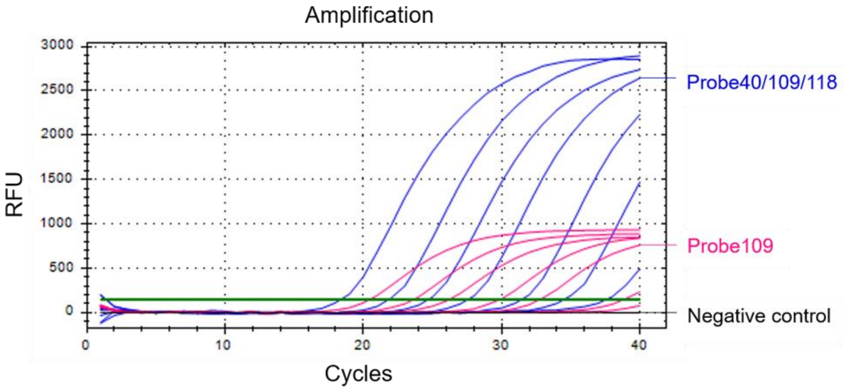

Fig. S1 Sensitivity test curves of single-probe 109 and triple-probe 40/109/118 TaqMan qPCR assay.

The dynamic curves were generated by using final concentration of the plasmid DNA ranged from 5.0×10^5 copies/μL to 5.0×10^{-1} copies/μL.
